# Persistent left superior vena cava as an incidental fnding in the introduction of a transient pacemaker: A case report

**DOI:** 10.7705/biomedica.6505

**Published:** 2022-09-02

**Authors:** David Ricardo Echeverry, Juan Guillermo Buitrago, Andrés Alirio Restrepo, Cristhian David Morales

**Affiliations:** 1 Departamento de Medicina Crítica, Hospital Universitario San Jorge de Pereira, Pereira, Colombia Hospital Universitario San Jorge de Pereira Pereira Colombia; 2 Departamento de Medicina Crítica, Facultad de Ciencias de la Salud, Universidad Tecnológica de Pereira, Pereira, Colombia Universidad Tecnológica de Pereira Universidad Tecnológica de Pereira Pereira Colombia; 3 EBM Developing Countries Foundation, Guadalajara de Buga, Colombia EBM Developing Countries Foundation Guadalajara de Buga Colombia; 4 Departamento de Urgencias y Atención Primaria, Comfandi IPS, Guadalajara de Buga, Colombia Comfandi IPS Guadalajara de Buga Colombia; 5 Grupo de Investigación en Farmacoepidemiología y Farmacovigilancia, Departamento de Ciencias Básicas, Universidad Tecnológica de Pereira, Pereira, Colombia Universidad Tecnológica de Pereira Universidad Tecnológica de Pereira Pereira Colombia; 6 Departamento de Salud Pública, Facultad de Salud, Universidad Autónoma de Manizales, Manizales, Colombia Universidad Autónoma de Manizales Universidad Autónoma de Manizales Manizales Colombia

**Keywords:** Vena cava, superior, incidental fndings, heart defects, congenital, echocardiography, acute coronary syndrome, percutaneous coronary intervention, case reports, vena cava superior, hallazgos incidentales, cardiopatías congénitas, ecocardiografía, síndrome coronario agudo, intervención coronaria percutánea, informes de casos

## Abstract

The persistent left superior vena cava is the most common venous anomaly in the systemic drainage in adults and tends to be asymptomatic. The persistent left superior vena cava causes rhythm disorders such as tachyarrhythmias or bradyarrhythmias.

We report a case of persistent left superior vena cava diagnosed in a 53-year-old female patient admitted due to an acute coronary syndrome associated with unstable bradycardia. A transvenous peacemaker impressed the left atrium; therefore, a transthoracic echocardiogram was required to diagnose persistent left superior vena cava. The patient needed management with percutaneous intervention; she had an adequate evolution and subsequent discharge from the intensive care unit

## Resumen

La vena cava superior izquierda persistente es la anomalía venosa más frecuente en el drenaje sistémico en adultos; tiende a ser asintomática, pero causa trastornos del ritmo como taquiarritmias o bradiarritmias.

Se presenta un caso de vena cava superior izquierda persistente diagnosticada en una paciente de 53 años hospitalizada por un síndrome coronario agudo asociado a bradicardia inestable. Un marcapasos transvenoso dejó una impresión en la aurícula izquierda, por lo que se requirió un ecocardiograma transtorácico para diagnosticar la vena cava superior izquierda persistente. La paciente necesitó una intervención percutánea con una adecuada evolución y se le dio el alta de la unidad de cuidados intensivos.

The persistent left superior vena cava is the most common variant of the systemic venous drainage in adults with an incidence of 0.3 to 0.5% in the general population and 4 to 8% in patients with congenital heart disease ^1^^-^^3^. This condition results from a failure to obliterate the left common cardinal vein typically draining into the left subclavian vein and jugular veins into the right atrium through the coronary sinus ^2^. Approximately, 20% of the total venous blood returns from the left arm and left half of the head and neck in persistent left superior vena cava. Right atrial drainage occurs in 80 to 90% of cases ^2^. 

This abnormality can be identifed incidentally on echocardiography as a dilatation of the coronary sinus through tomography or resonance imaging as a vessel that runs vertically from the lateral mediastinum to the aortic arch ^2^^,^^4^. In most individuals with persistent left superior vena cava, many cardiac anomalies may be identified: right-sided lesions, single ventricular abnormalities, left-side obstructive lesions, conotruncal malformations, shunt lesions, and aortic arch anomalies ^4^. However, persistent left superior vena cava does not usually have clinical repercussions unless it drains the left cavities ^2^^,^^4^.

The relationship of persistent left superior vena cava with intracardial, extracardiac, and chromosomal abnormalities in fetuses has been elucidated in a systematic review ^5^ while different clinical reports have studied it in adult and pediatric populations, as well as its absence ^6^. Therefore, describing this defect may contribute to new findings with implications for clinicians who place central venous devices, especially in adults. Given that the right access to the heart is more complex, implanting pacemakers up on persistent left superior vena cava patients may generate incorrect positioning.

## Case report

A 53-year-old female patient with a history of heavy smoking was admitted to the emergency room due to oppressive chest pain, diaphoresis, and syncope associated with unstable sinus bradycardia and elevated troponin.

**Figure 1:**
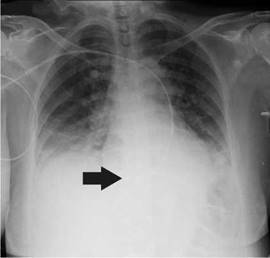
Radiographic control in the intensive care unit after the placement of the transvenous pacemaker. The arrow shows the end of the vascular access located in the left atrium.

**Figure 2:**
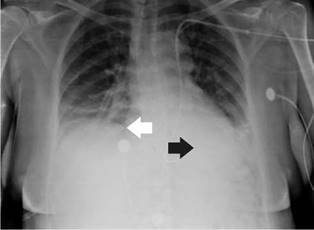
Radiographic control after new placement of transvenous pacemaker through the left subclavian access. The black arrow shows the end of the vascular access located in the right ventricle. The white arrow shows the right atrium.

She was diagnosed with cardiogenic shock secondary to non-ST elevation acute myocardial infarction and sinus bradycardia. The initial management in the emergency room, therefore, included dual antiplatelet therapy, anticoagulation, norepinephrine, and dopamine. Then, the patient was transferred to the intensive care unit where a transvenous pacemaker was implanted using the right subclavian anatomical approach with adequate return and no suitable capture. A control chest radiography was performed which revealed no pneumothorax and hemothorax but evidenced the extreme of the pacemaker on the left atrium (figure 1). The pacemaker was considered to be poorly located, so a new one was placed through the left subclavian anatomical approach without complications and adequate capture. However, the team suspected a vascular malformation in the control chest radiograph because the extreme part of the pacemaker was located on the right ventricle (figure 2).

**Figure 3:**
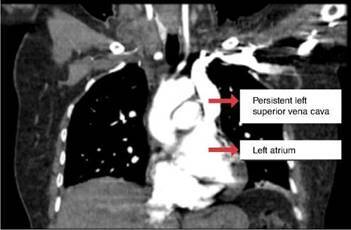
Chest computed tomography angiography showing left superior vena cava draining abnormally into the left atrium

An echocardiogram done showed coronary sinus dilation, which suggests persistent left superior vena cava with the electrode in the coronary sinus dilated. The computed tomography (CT) angiogram showed a superior vena cava on the left that drained abnormally on the left atrium communicating with the right atrium (figure 3). Subsequently, a left cardiac catheterization was performed in another institution on the patient due to myocardial infarction.

## Discussion

The high incidence of persistent left superior vena cava usually responds to coexisting issues in these patients, such as congenital or rhythm abnormalities, requiring the placement of pacemakers due to the embryonic origin of pacemaker cells derivated near the root of the superior vena cava. Normal embryonic development on the right located in the venous sinus usually creates the sinus node ^7^^,^^8^. The left posterior cardinal vein migrates toward the coronary sinus. The tissue then loses the conduction capacity when the vein degenerates but it does not obliterate and persistent left superior vena cava is developed ^7^^,^^8^. Thus, persistent left superior vena cava generates tachyarrhythmias and bradyarrhythmias, especially when the catheterization or electrode pitch is done. Furthermore, there are cases of cardiac arrest, shock, angina, coronary sinus perforation of the brachycephalic vein, or even death due to these procedures in patients with this anomaly ^7^^,^^8^. Also, it is crucial to notice that stress factors may influence the genesis of arrhythmias in the conduction tissue, such as suitable atrium growth or coronary sinus dilation ^9^, as was the case in this patient.

The persistent left superior vena cava remains asymptomatic because it does not cause a right-to-left shunt; however, in some cases, this shunt is pronounced because of desaturation ^4^. It manifests itself with cyanosis, reduced exercise tolerance, progressive fatigue, and syncope ^4^, some of which were present in our patient. Likewise, these patients may develop brain abscesses and thromboembolic events; in this case, persistent left superior vena cava should be ligated or may be re-anastomosed to the coronary sinus ^4^.

Most of the cases of persistent left superior vena cava are discovered when difficulties arise during invasive procedures such as cardiac resynchronization therapy, central venous catheter insertion, or pacemaker implantation ^4^^,^^10^. The persistent left superior vena cava could complicate the implantation of the pacemaker by causing fixation difficulties of the electrode because of the tortuous course ^3^^,^^4^. The central venous catheter insertion may generate angina, hypotension, and heart perforation ^4^. Catheterization may be challenging and may change to the proper access. However, complications such as tamponade, cardiogenic shock, and arrhythmias can appear ^4^^,^^11^.

Appropriate entry is difficult in some cases when there is no right superior vena cava ^4^.In the literature, there are various cases with successful intracardiac device implants using the left approach ^3^^,^^4^^,^^11^. Different diagnostic methods such as transthoracic echocardiogram or transesophageal echocardiogram, conventional and cross-sectional venography and computed tomography (CT) venography are used to determine persistent left superior vena cava ^12^. Thus, CT venography may detect persistent left superior vena cava in 2.6% of patients with the dilation of coronary sinus being more frequent than other findings ^13^.

Generally, the presence of persistent left superior vena cava does not require surgical management, however, this approach can be considered in patients with anomalous drainage in the left atrium. Different surgical techniques may correct this abnormality depending on the anatomical characteristics of the patients, for example, reimplanting the left superior vena cava in the right atrium ^14^.

In our case, the patient presented intracardiac conduction abnormalities in the context of an acute coronary event. The incidental finding of persistent left superior vena cava was revealed by chest radiography showing the positioning of the transvenous pacemaker electrodes. Likewise, the patient presented coronary sinus dilation, one of the findings reported in the literature ^4^. However, our patient presented anomalous drainage of the vena cava superior to left cavities, which is unusual in most clinical reports. It is also noteworthy that the patient initially attempted the approach through the convenient internal jugular access without success and finally through the left subclavian access. This allowed the pacemaker to capture the rhythm and is the preferred approach in the case of isolated persistent left superior vena cava.

## Conclusions

The persistent left superior vena cava is not an infrequent clinical finding. It is important, therefore, that the clinician acquire adequate knowledge regarding this vascular abnormality because these anatomical variants can result in unconventional paths observed in chest radiographs. It generates erroneous conclusions for the decisions regarding the placing of different intravascular devices as in certain circumstances, changing the device is unnecessary. In cases such as ours, draining into the left atrium may generate clinical repercussions such as arrhythmias and bradyarrhythmias, although this finding is not frequent.
